# Functional Nanostructured Oligochitosan–Silica/Carboxymethyl Cellulose Hybrid Materials: Synthesis and Investigation of Their Antifungal Abilities

**DOI:** 10.3390/polym11040628

**Published:** 2019-04-04

**Authors:** Thuy N Nguyen, Thu NM Huynh, DongQuy Hoang, Dai Hai Nguyen, Quoc Hien Nguyen, Thai Hoa Tran

**Affiliations:** 1 Department of Polymer and Composite Materials, Faculty of Materials Science and Technology, University of Science, Vietnam National University, Ho Chi Minh City 700000, Vietnam; nnthuy@hcmus.edu.vn (T.N.N.); huynhminhthu1204@gmail.com (T.N.M.H.); 2 Institute of Applied Materials Science, Vietnam Academy of Science and Technology, 01 TL29, District 12 Ho Chi Minh City 700000, Vietnam; nguyendaihai0511@gmail.com; 3 Research and Development Center for Radiation Technology, Viet Nam Atomic Energy Institute, Ho Chi Minh City 700000, Vietnam; hien7240238@yahoo.com; 4 College of Sciences, Hue University, Hue City 530000, Vietnam

**Keywords:** oligochitosan, nanosilica, hybrid material, antifungal agent, *Phytophthora infestans*

## Abstract

Functional hybrid materials were successfully synthesized from low-cost waste products, such as oligochitosan (OCS) obtained from chitosan (one of the main components in crab shells) and nanosilica (nSiO_2_) obtained from rice husk, in a 1:1 ratio (*w*/*w*), and their dispersion in the presence of carboxymethyl cellulose at pH 7 was stable for over one month without aggregation. The molecular weights, chemical structures, morphologies, and crystallinities of the obtained materials were characterized by GPC, FTIR, TEM, and XRD, respectively. The antifungal effects of OCS, nSiO_2_, and the OCS/nSiO_2_ hybrid materials were investigated via a disk-diffusion method. The results showed that the nanohybrid materials had better resistance to *Phytophthora infestans* fungus than the individual components, and a concentration of the OCS2/nSiO_2_ hybrid material of 800 mg L^−1^ was the lowest concentration where the material completely inhibited *Phytophthora infestans* growth, as measured via an agar dilution method. This study not only creates a novel environmentally friendly material with unique synergistic effects that can replace current toxic agrochemicals but also can be considered a new platform for further research in green agricultural applications.

## 1. Introduction

Chitosan is the second most abundant natural polysaccharide and has a chemical structure that includes glucosamine and acetyl glucosamine monomer units linked via β (1–4) glycosidic bonds. Chitosan has a variety of unique functional characteristics, such as biodegradability, biocompatibility, nontoxicity, antibacterial, and antifungal properties; as such, it can be applied to a number of fields. Nonetheless, its poor solubility limits its utility in several applications, such as in food, biomedicine and agricultural fields [1]. In contrast to chitosan, oligochitosan (OCS), which is obtained by the degradation of chitosan, has short chain lengths, low viscosity, free amino groups, and great solubility at a neutral pH in addition to superior properties to those mentioned above for chitosan, resulting in it receiving increasing attention from many researchers recently [2,3,4].

Silica is a type of mineral that consists of silicon and oxygen, which are the two most abundant elements in the crust of the earth. Although it is regarded as a necessary nutrient source that plays an important role in stimulating the growth of plants as well as supporting the self-defense response of plants against various diseases, silica mostly occurs in the crystalline state and rarely in an amorphous state in nature. Therefore, plants rarely absorb natural silica, while synthesized silica, notably nanosilica (nSiO_2_), used for agricultural purposes is in the amorphous form. nSiO_2_ has been found to help improve soil properties and increase the germination rate of seeds [5] as well as reduce the germination time of tomato seeds, while significantly acting on pest and disease resistance in plants, which has led to increased yields in a number of crops [6,7].

*Phytophthora infestans* (*P. infestans*) is a heterothallic oomycete, which is a near-obligate hemibiotrophic pathogen under natural and agricultural conditions. Its asexual cycle enables incredibly rapid population growth in susceptible host tissue, and sporangia are produced on sporangiophores that grow from infected tissue [8]. Over 150 years after it was first discovered that late blight disease was caused by *P. infestans* in Europe and in North America [9], it still remains a major problem in agriculture and a dangerous pathogen causing serious decreases in crop yield, and can even drive modern farmers out of business, especially in Vietnam, where there is no stable disease suppression, and safe and effective treatments have not been available. This has resulted in commercially available toxic agrochemicals, such as metalaxyl and antifungal antibiotics, being widely marketed with little regulation, and the improper use of these fungicides for the early control of late blight disease has led to not only environmental pollution but also hazardous influences on human health. Simultaneously, the application of fungicides has brought about the emergence of fungicide-resistant strains of fungi.

The development of OCS-nSiO_2_ hybrid materials dispersed in carboxymethyl cellulose (CMC) as effective eco-friendly agrochemicals from the abundant waste resources of agriculture and aquaculture is meaningful and valuable for solving the alarming problems mentioned above. It was hypothesized that when the nSiO_2_ particle is small, the more silica particles accumulate in the cell membrane, the cell wall of fungi can be easily broken. Furthermore, OCS products with small molecular weights will show higher antifungal ability than that of chitosan. And therefore, it is assumed that the combination of OCS and nSiO_2_ in the presence of CMC creates new hybrid materials having a synergistic resistance effect against P. infestans. The inhibition zones of the hybrid materials are expected to be much larger than those of the individual components at the same concentrations. To the best of our knowledge, until now, no publications that study the synthesis of the nanostructured oligochitosan-silica hybrid materials with carboxymethyl cellulose stabilizer and investigate their antifungal abilities has been reported. Thus, this study, which is the first investigation on the preparation of an environmentally friendly material with unique synergistic effects between OCS and nSiO_2_ with CMC stabilizer that can replace toxic agrochemicals, has applications in the current situation. The synthesis and investigation of the fungicidal ability of different kinds of OCS-nSiO_2_ hybrid materials prepared from OCS with different molecular weights are also discussed. Building on the outcomes of this current study, the possible antifungal mechanisms are also described in this report.

## 2. Materials and Methods

### 2.1. Materials

Chitosan, derived from chitin with a 90.46% degree of deacetylation (DD) and a molecular weight (*M*_w_) of 94.28 kDa, was supplied by the VINAGAMMA center (Ho Chi Minh, Vietnam). Raw rice husks (RHs) were bought in southern Vietnam. H_2_O_2_ 30% (d: 1.11 g mL^−1^) was purchased from Merck (Darmstadt, Germany). All other chemicals, including carboxymethyl cellulose (CMC), with a *M*_w_ of 557.79 kDa, lactic acid, sodium hydroxide, hydrochloric acid, ammonium hydroxide and ethyl alcohol, were of reagent grade and obtained from JHD Chemical (Xilong, China). Distilled water was used in all preparations. The *Phytophthora infestans* (*P. infestans*) fungus was provided by the Institute of Applied Materials Science, Vietnam Academy of Science and Technology ( Ho Chi Minh, Vietnam).

### 2.2. Preparation of Oligochitosan (OCS) with Different Molecular Weights

Three categories of OCS, including OCS1 (*M*_w_ = 5.48 kDa), OCS2 (*M*_w_ = 4.21 kDa), and OCS3 (*M*_w_ = 3.60 kDa), were obtained through the heterogeneous degradation of the initial chitosan by H_2_O_2_ (1 w v^−1^) prior to homogeneously degrading chitosan by irradiation a 4% (w v^−1^) chitosan/1% (w v^−1^) H_2_O_2_ solution in a gamma SVST Co-60/B irradiator at an absorbed dose of 12 kGy, 17 kGy and 27 kGy, respectively [10].

### 2.3. Preparation of Nanosilica (nSiO_2_) from the Rice Husk

Rice husks (RHs) were washed with water to remove dust, soluble substances, and other contaminants before being dried at 60 °C in a forced air oven. Then, 50.0 g of dried RHs was treated with 800 mL of a 1% (w w^−1^) HCl solution at room temperature for 2 h by magnetic stirring and kept overnight before decanting, and thoroughly washing with distilled water. Then, the acid-treated RHs were incinerated at 700 °C for 2 h inside a programmable furnace to obtain nSiO_2_.

### 2.4. Preparation of Hybrid Materials

The synthesis of hybrid materials based on OCS and nSiO_2_ with a mass ratio of 1:1 was carried out as previously reported [11,12] with modifications as follows. nSiO_2_ (1.00 g) was dissolved in 6.65 mL of 1 M NaOH in beaker A, while CMC (0.30 g) was dissolved with distilled water in beaker B. Afterwards, the CMC solution was poured into beaker A and stirred for 2 h. The obtained solutions of OCS with different molecular weights (OCS1, OCS2, and OCS3) were slowly added dropwise into the mixture in beaker A to create OCS1/nSiO_2_, OCS2/nSiO_2_, and OCS3/nSiO_2_, respectively, and the pH values of these mixtures were adjusted to 7 by 1 M HCl. They were stirred for 3 h prior to being stored at room temperature.

### 2.5. Characterization and Measurements

The average molecular weights of the chitosan samples were measured by an Agilent GPC (LC-20AB; Shimadzu, Kyoto, Japan) with an RI-10A detector and 250 ultrahydrogel column from Waters (Milford, MA, USA) together with an aqueous solution of 0.25 M CH_3_COOH/0.25 M CH_3_COONa as the eluent at a flow rate of 1 mL min^-1^.

The chemical structures of OCS, CMC, nSiO_2_, and OCS/nSiO_2_ were analyzed by using an FTIR 8400S spectrometer (Shimadzu, Kyoto, Japan) and KBr pellets. The DD of the chitosan samples was calculated based on FTIR spectra according to the following equation:

DD = 100 − [(A_1320_/A_1420_ − 0.3822)/0.03133] (1)

where A_1320_ and A_1420_ are absorbances of chitosan at 1320 and 1420 cm^− 1^, respectively.

The X-ray diffraction (XRD) pattern of nSiO_2_ was recorded on an X-ray diffractometer, D8 Advance A25 (Brucker, Karlsruhe, Germany) in the scattering range (2θ) of 0°–80° with a step rate of 0.25 °/min. The particle sizes and morphologies of nSiO_2_ and the OCS/nSiO_2_ samples were investigated using transmission electron microscopy on a JEM1400 (JEOL, Tokyo, Japan).

### 2.6. Antifungal Effect Test on Phytophthora Infestans

The antifungal activities of chitosan, oligochitosan, silica, and the hybrid materials were investigated via a paper disk-diffusion method. First, carrot glucose agar plates (CGA, carrot infusion 220 g L^−1^, glucose 20 g L^−1^, agar 9 g L^−1^) were prepared as described in a previous report [13] with several modifications. Second, cell suspensions obtained from fungal colonies were spread directly on the agar surface. Then, paper discs (diameter 6 mm) loaded with a volume (50 µL) of the antimicrobial agents at different concentrations (600, 800, 1000, 1200, 1400, 1600, and 1800 mg L^−1^) in distilled water were dried in air and placed on the inoculated agar plates and incubated for two days. The inhibition zones were calculated from their diameters. The statistics were analyzed by using two-way analysis of variance (ANOVA) with *p*-values less than 0.05 being considered statistically significant.

### 2.7. Investigation of the Minimum Inhibitory Concentration (MIC)

According to the standard agar dilution method [14], the preparation was as follows: an appropriate dilution of a OCS2/nSiO_2_ solution was added to prepared molten test agars to ensure that the concentrations of OCS2/nSiO_2_ in the mixtures were 400, 600, 800, 1000, 1200, 1400, 1600, and 1800 mg L^−1^. Then, the mixtures were poured into Petri dishes (90 mm × 15 mm). Inoculum samples were spread on the plates and fungi were grown for 10 days. Antifungal agent-free plates were used as control plates, and the tests were repeated three times. The MIC values were evaluated based on the lowest concentrations of antifungal agents, where no growth of *P. infestans* was observed.

## 3. Results and Discussion

### 3.1. Characterization of the Oligochitosan

**Figure 1:**
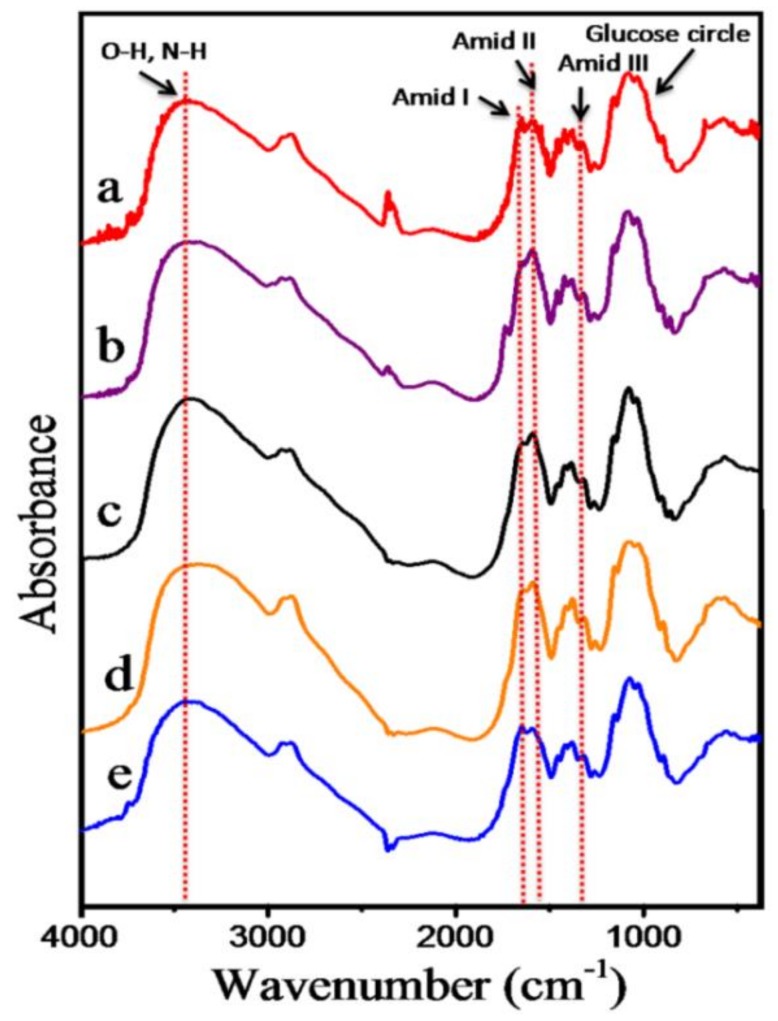
FTIR spectra of the initial chitosan (CTS0, *M*_w_ = 94.28 kDa) (**a**), chitosan obtained through the heterogeneous degradation of chitosan by H_2_O_2_ (CTS1, *M*_w_ = 48.35 kDa) (**b**), OCS1 (*M*_w_ = 5.48 kDa) (**c**), OCS2 (*M*_w_ = 4.21 kDa) (**d**), and OCS3 (*M*_w_ = 3.60 kDa) (**e**).

The FTIR spectra of the original chitosan and the oligochitosan products produced by the irradiation with different doses of chitosan in the presence of 0.5% H_2_O_2_ are displayed in Figure 1. The FTIR spectrum of the initial chitosan possesses characteristic absorption peaks, including those at 3200–3500 cm^−1^ (the stretching vibrations of O–H and N–H), 2888 cm^−1^ (the stretching vibration of C–H), and a range of peaks from 1159–896 cm^−1^ (the stretching vibrations of C–O and C–O–C in the glucose ring). The peaks at 1648 cm^−1^ (amide I), 1586 cm^−1^ (amide II), 1420 cm^−1^ (the symmetrical deformations of –CH_3_ and –CH_2_), and 1320 cm^−1^ (the absorbance of the C–N in CH_3_CONH–, amide III) were calculated to define the DD of chitosan. Additionally, the FTIR spectra of the irradiated products did not differ from the FTIR results of the initial chitosan; notably, the main functional groups of these materials are still present, and there are no structural changes in chitosan after irradiation. The DDs of CTS0, CTS1, OCS1, OCS2, and OCS3 were 90.46, 88.2, 87.88, 84.27, and 86.42, respectively.

### 3.2. Characterization of the Nanosilica

**Figure 2:**
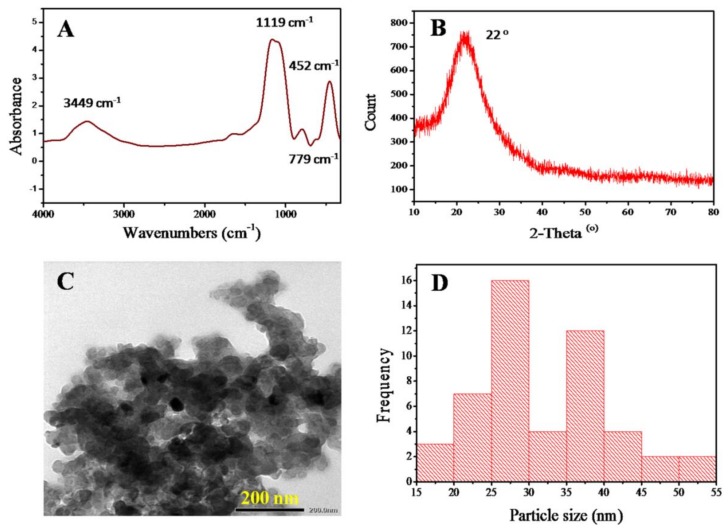
(**A**) FTIR spectrum, (**B**) XRD pattern, (**C**) TEM micrograph, and (**D**) diagram of the particle size distribution of the nSiO_2_ obtained from raw rice husks.

After the incineration process of acid-treated RHs at 700 °C, the obtained nSiO_2_ was analyzed by FTIR, XRD, and TEM. Figure 2A shows that characteristic peaks appear at 1119 cm^−1^ (the asymmetrical stretching vibration of O–Si–O), 779 cm^−1^ (the symmetrical stretching vibration of O–Si–O), and 452 cm^−1^ (the bending vibration of O–Si–O) [15]. The band at 1179–1200 cm^−1^ is attributed to the asymmetric stretching mode of the SiO_4_ coordination units; the broad peak at 3449 cm^−1^ is assigned to stretching of the –OH groups, while a small and low peak that appeared at 1639 cm^−1^ is related to the bending vibration of water molecules absorbed onto the surface of the silica particles [11,16].

Figure 2B shows the XRD pattern of the nSiO_2_ sample derived from the combustion of acid-treated RH powders at 700 °C for 2 h. It shows that there is only one broad peak at 2θ = 22°, indicating that the generated nSiO_2_ was pure and had an amorphous structure, which is similar to the results of previous reports [17].

The particle morphology and size of the obtained nSiO_2_ after the combustion process were analyzed via TEM and are displayed in Figure 2C,D. The TEM image and particle size distribution graph show that the shape of the nSiO_2_ particles is nearly spherical, their average size is approximately 25–40 nm, and they have a tendency to form clusters during synthesis.

### 3.3. Characterization of the Hybrid Materials

The three categories of obtained hybrid materials include OCS1/nSiO_2_, OCS2/nSiO_2_, and OCS3/nSiO_2_, and they were created by interactions between the nSiO_2_ obtained from RHs and OCS samples with different *M*_w_ of 5.48, 4.21, and 3.60 kDa in a 1:1 ratio. To stabilize the hybrid materials during long-term storage, CMC, at a concentration of 0.3% (*w*/*v*), was added to the mixture; that is, CMC can help minimize the precipitation phenomenon of the hybrid materials.

**Figure 3:**
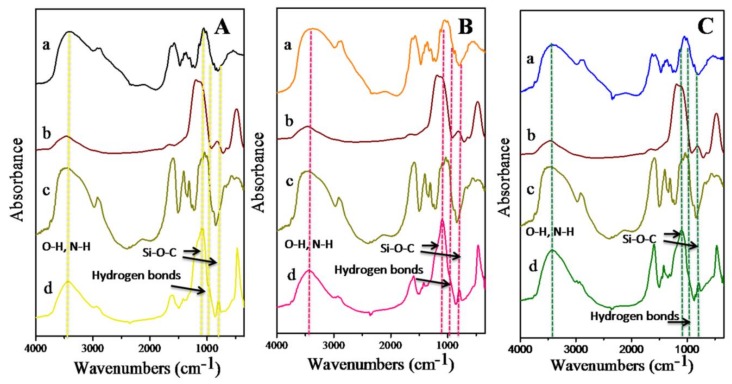
FTIR spectra of OCS1/nSiO_2_ (**A**(d)), OCS2/nSiO_2_ (**B**(d)), and OCS3/nSiO_2_ (**C**(d)) as compared to the FTIR spectra of OCS1 (**A**(a)), OCS2 (**B**(a)), COS3 (**C**(a)), nSiO_2_ (**A**(b), **B**(b) and **C**(b)), and CMC (**A**(c), **B**(c) and **D**(c)).

The observed FTIR spectrum of CMC reveals a strong broad peak at 3460 cm^−1^ (the stretching vibration of –OH) and a peak at 2923 cm^−1^ (the stretching vibration of C–H) (Figure 3). Two strong peaks at 1608 and 1421 cm^−1^ can be observed due to the appearance of the asymmetrical and symmetrical stretching, respectively, of the –COO– groups [18,19]. Moreover, a peak at 1322 cm^−1^ is attributed to the bending vibrations of the –CH_2_ and –CH_3_ groups, and a peak at 1054 cm^−1^ is assigned to the >CH–O–CH_2_ group [20].

In comparison with the FTIR spectra of nSiO_2_ and OCS, Figure 3A(d),B(d),C(d) show that the FTIR spectra of the hybrid materials possess the characteristic absorption peaks of both OCS and nSiO_2_. However, the bands of the O–H and N–H stretching vibrations of OCS at 3400 cm^−1^ are narrower and shifted to higher wavenumbers in the FTIR spectra of the hybrid materials than in the OCS spectrum due to the interactions of OCS with nSiO_2_. New bands appear at 1083 cm^−1^ and 799 cm^−1^ (the vibrations of the newly created Si–O–C bond) in the FTIR spectrum of the OCS1/nSiO_2_ hybrid material. Additionally, a shoulder peak at 971 cm^−1^ can be assigned to the formation of hydrogen bonds between the silanol groups on the surface of silica and the amid and oxy groups of OCS/CMC [21], while the amide I band of OCS1 at 1642 cm^−1^ shifts to 1622 cm^−1^ and the position of the amide II peak at 1589 cm^−1^ also relocates to 1598 cm^−1^ in the FTIR spectrum of OCS1/nSiO_2_, which indicates the appearance of associations between OCS and CMC. Moreover, a weak shoulder peak at 1735 cm^−1^ reflects the interactions between the –COOH groups of CMC and the –NH_2_ groups of OCS [19].

Regarding the OCS2/nSiO_2_ hybrid material, there were only small differences in the characteristic peak positions in comparison with the FTIR spectrum of the OCS1/nSiO_2_ hybrid material. Figure 3B(d) shows a small shoulder peak at 963 cm^−1^ (due to the formation of hydrogen interactions between nSiO_2_ and OCS/CMC) in the FTIR spectrum of OCS2/nSiO_2,_ while these interactions appear as a peak at 971 cm^−1^ in the FTIR spectrum of OCS1/nSiO_2_. The appearance of peaks at 1105 and 801 cm^−1^ indicates the existence of the Si–O–C bond. Additionally, OCS2/nSiO_2_ has shifts of the characteristic absorption peaks, notably, the amide I band at 1642 cm^−1^ of OCS2 shifted to a higher wavenumber of 1643 cm^−1^ instead of a lower wavenumber (1622 cm^−1^), as in the case of OCS1/nSiO_2_, and the amide II peak shifted from 1592 to 1598 cm^−1^ with a higher intensity in the FTIR spectrum of OCS2/nSiO_2_ than in that of OCS1/nSiO_2_.

Likewise, Figure 3C shows that OCS3/nSiO_2_ created from the combination of nSiO_2_ and OCS3 in the presence of CMC has shifts in the characteristic peaks toward higher wavenumbers (amide I (1648 cm^−1^) and amide II (1595 cm^−1^)) and the new peaks at 1108 cm^−1^ and 781 cm^−1^ can be attributed to the association between the components.

**Figure 4:**
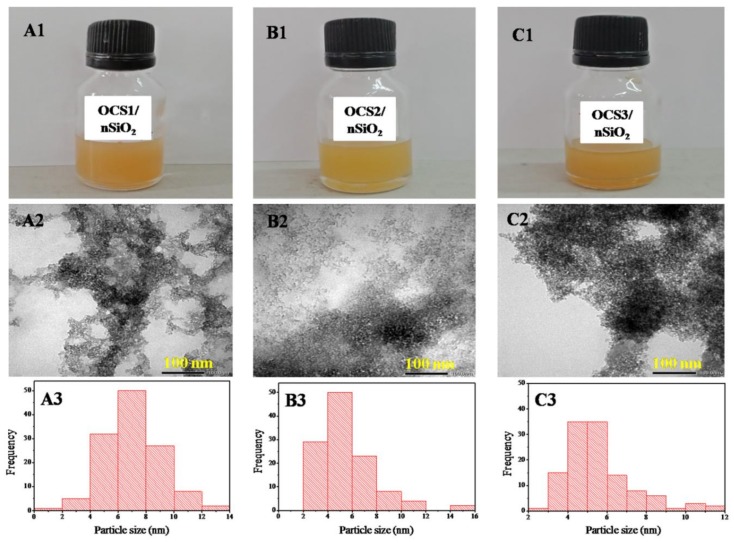
Photographs of the hybrid materials, TEM micrographs, and diagrams of particle size distributions for OCS1/nSiO_2_ (**A**(1), **A**(2), and **A**(3)), OCS2/nSiO_2_ (**B**(1), **B**(2), and **B**(3)), and OCS3/nSiO_2_ (**C**(1), **C**(2), and **C**(3)), respectively.

Figure 4A(1),B(1),C(1) show the produced hybrid material solutions stored at room temperature for 24 h without the appearance of aggregation. These OCS1/nSiO_2_, OCS2/nSiO_2_, OCS3/nSiO_2_ hybrid samples were analyzed via TEM to investigate their morphologies (Figure 4A(2),B(2),C(2)) as well as particle sizes (Figure 4A(3),B(3),C(3)). The results indicate that the particle sizes of the hybrid materials are far smaller than that of the nSiO_2_ obtained from RH. In particular, the particle size of the OCS1/nSiO_2_ sample is 4–8 nm in diameter, while the particle sizes of the OCS2/nSiO_2_ and OCS3/nSiO_2_ samples are approximately 2–8 nm and 3–7 nm, respectively. This finding can be explained by the first phase of the synthetic process for the OCS/nSiO_2_ hybrid materials, in which silica becomes sodium silicate in the base medium. Then, oligochitosan/carboxymethyl cellulose plays a role in stabilizing the silica particles that are regenerated during the reaction of the sodium silicate and OCS/CMC solution. This helps prevent the nSiO_2_ particles from aggregating. However, the different particle size ranges of the three different hybrid materials may be related to the formation abilities of the interactions between the components.

### 3.4. Antifungal Effect Tests on Phytophthora Infestans

**Figure 5:**
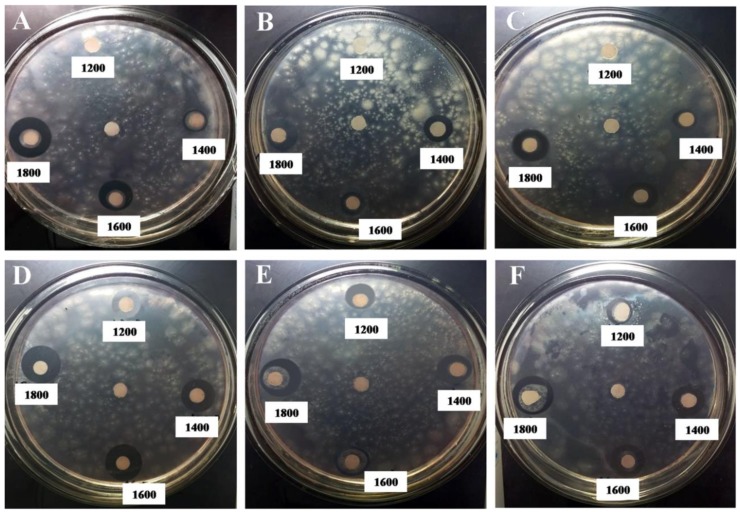
Images of the observed inhibition zones of OCS1 (**A**), OCS2 (**B**), OCS3 (**C**), OCS1/nSiO_2_ (**D**), OCS2/nSiO_2_ (**E**), and OCS3/nSiO_2_ (**F**) against *P. infestans*. A blank control without any antifungal agents was placed at the center of each plate.

Chitosan (CTS1), oligochitosan (OCS1, OCS2, and OCS3), nSiO_2_ and the hybrid samples (OCS1/nSiO_2_, OCS2/nSiO_2_, and OCS3/nSiO_2_) were investigated for their antifungal abilities against *P. infestans* via a paper disc diffusion method, and the measured results are displayed in Table 1 and Figure 5.

When CTS1 with a high molecular weight (*M*_w_ = 48.35 kDa) was used as the antifungal agent at a concentration of 1200 mg L^−1^, the diameter of the inhibition zone that begins to appear is approximately 6.83 ± 0.17 mm. However, despite increasing the concentration of CTS1 from 1200 to 1800 mg L^−1^, this diameter is nearly unchanged, approximately 6.67–7.17 mm, which indicates that its antifungal activity remained essentially constant. Additionally, the OCS products with small molecular weights showed antifungal effects at 1400 mg L^−1^. Although the fungal inhibition starts to appear at a higher concentration than that of CTS1, the inhibitory zone diameters at 1400 mg L^−1^ for the OCS1, OCS2, and OCS3 samples are 1.16, 2.60, and 3.00 mm, respectively, greater than that of CTS1 at the same concentration. Moreover, the diameters of the inhibition zones had an increasing trend when the concentration of the OCS antifungal agents increased from 1400 to 1800 mg L^−1^ (Figure 5). OCS3, which has the smallest molecular weight (*M*_w_ = 3.60 kDa), showed the highest antifungal ability among the three OCS samples based on the diameters of its inhibition zones at all investigated concentrations always being larger than those of OCS1 (*M*_w_ = 5.48 kDa) and OCS2 (*M*_w_ = 4.21 kDa).

These results indicate that the smaller the molecular weight of chitosan, the higher the antifungal ability of chitosan. Similar to paromomycin or other aminoglycoside antibiotics, the primary mechanism of the cytotoxicities of chitosan and oligochitosan can be related to mitochondrial mutations leading to 12S rRNA binding with a higher affinity to the aminoglycosides, which can cause misreading of the genetic code and mistranslated proteins in the fungi [22]. However, the antifungal activity of OCS is better than that of chitosan. It can be reasoned that compared to chitosan with a high molecular weight, the low molecular weight OCS is more readily absorbed into the microbial cells of fungi [23], and simultaneously, the intramolecular hydrogen bonds and van der Waals forces in the short OCS chains are drastically reduced during the preparation of OCS, as mentioned in our previous report [10]; that is, the hydroxyl and amino groups are more active, which plays a primary role in the antifungal activity of OCS. The growth and normal physiological functions of a fungal pathogen can be directly disrupted by the formation of polyelectrolytic complexes between the positively charged amino groups of OCS and the negatively charged groups on the cell surface, which can lead to disordering of the cell surface [23,24].

In terms of nSiO_2_, the results of the investigation on its antifungal capability in the concentration range of 600 to 1800 mg L^−1^ show the appearance of an inhibition zone against *P. infestans* once the concentration reached 1200 mg L^−1^, as in the case of CTS1, and the diameter of the inhibition zone was approximately 7.77 ± 0.15 mm, which is approximately 0.94 mm larger than that of CTS1 at the same concentration (Table 1). When the concentration of nSiO_2_ increased from 1400 to 1800 mg L^−1^, the inhibition zone diameter slightly expands from 8.00 ± 0.00 mm to 9.77 ± 0.15 mm. However, the inhibition zone diameters of nSiO_2_ are smaller in comparison with those of the OCS samples at 1400–1800 mg L^−1^. The different antifungal activities of nSiO_2_ and OCS may depend on their fungal inhibition mechanisms. Until now, there has not been specific research on the mechanism of nSiO_2_ inhibition against *P. infestans*. According to earlier reports [25,26,27,28], there are several theories in this regard, which are as follows. The antifungal activity of nSiO_2_ may be related to protein molecule deactivation as well as direct interaction with the DNA of the fungal pathogen leading to DNA mutations and replication damage. In addition, the cell wall of fungi can be easily broken when the nSiO_2_ particles are small and the hydroxyl groups on their surface easily create hydrogen links with the lipopolysaccharides of the cell. As more silica accumulates in the cell membrane, it can bring about cell lysis because of its prevention of transmembrane energy cycling. In addition, insoluble substances can disrupt the electron transport chains fungal membrane or cause oxidation.

Regarding the hybrid materials, interestingly, whereas the lowest concentrations of nSiO_2_ and OCS to produce a fungal inhibitory effect against *P. infestans* were 1200 and 1400 mg L^−1^, respectively, the new nanostructured oligochitosan-silica hybrid materials showed antifungal abilities at a concentration of 800 mg L^−1^ with inhibition zone diameters of 10.17 ± 0.17 (OCS1/nSiO_2_), 9.67 ± 0.17 (OCS2/nSiO_2_), and 8.00 ± 0.50 mm (OCS3/nSiO_2_). Notably, the inhibition zones of all three hybrid materials were far larger than those of the individual components at the same concentrations. These results demonstrated our above assumption. The lowest concentration to prevent the growth of *P. infestans* in this investigation decreases by more than 1.5 times when OCS/nSiO_2_ hybrid materials are used as the antifungal agents, which proves that the combination of silica and oligochitosan successfully created a new material having much better antifungal activity against *P. infestans* than each individual substance.

The inhibitory zones of the three hybrid materials tended to significantly increase when their concentrations increased from 800 to 1800 mg L^−1^. During the investigation, the antifungal ability of OCS1/nSiO_2_ is the best, followed by that of OCS2/nSiO_2_, while OCS3/nSiO_2_ has the lowest antifungal capability. Although the antifungal activity of OCS3 against *P. infestans* is the best among the three surveyed oligochitosan samples, the hybrid material combining nSiO_2_ and OCS3 does not have the best antifungal synergistic effect. This phenomenon can be explained by the *M*_w_ of OCS3 being too small to fully coat the silica particles and create a stable hybrid material, such as OCS2/nSiO_2_ (the most stable mixture) and OCS1/nSiO_2_ (the second most stable hybrid material); the interaction forming abilities among the components of the OCS3/nSiO_2_ mixture can be considered to be lower than those of OCS1/nSiO_2_ and OCS2/nSiO_2_. This low ability can impact the antifungal effect of OCS3/nSiO_2_ and make it lower than that of OCS1/nSiO_2_ and OCS2/nSiO_2_; however, importantly, it still has a better antifungal effect than that of nSiO_2_ or OCS3.

**Figure 6:**
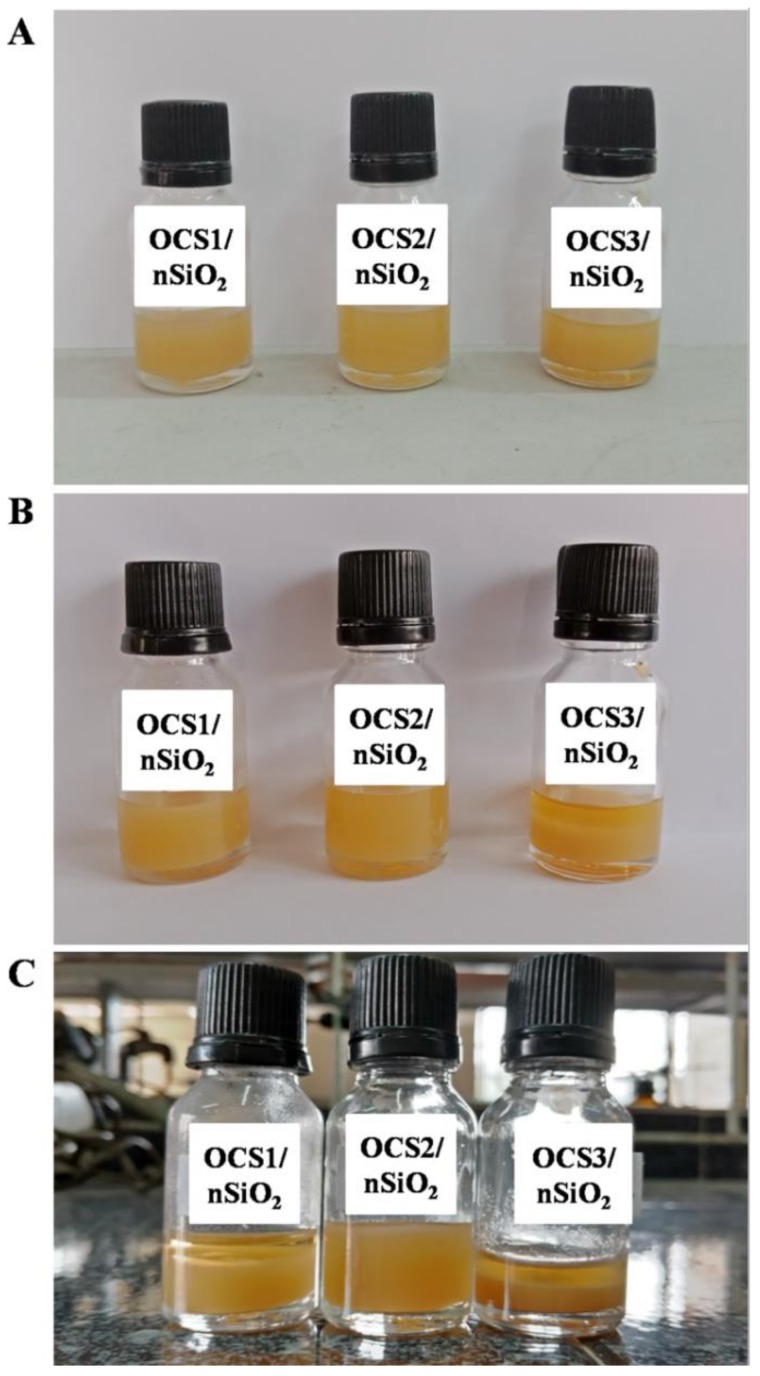
Images of OCS1/nSiO_2_, OCS2/nSiO_2_, and OCS3/nSiO_2_ after 1 day (**A**), 15 days (**B**), and 30 days (**C**).

As mentioned above, OCS2/nSiO_2_ is the most stable mixture among all three hybrid materials, showing no aggregation for more than one month when observing their stabilities (Figure 6), and simultaneously has good antifungal capability as opposed to that of OCS3/nSiO_2_.

**Figure 7:**
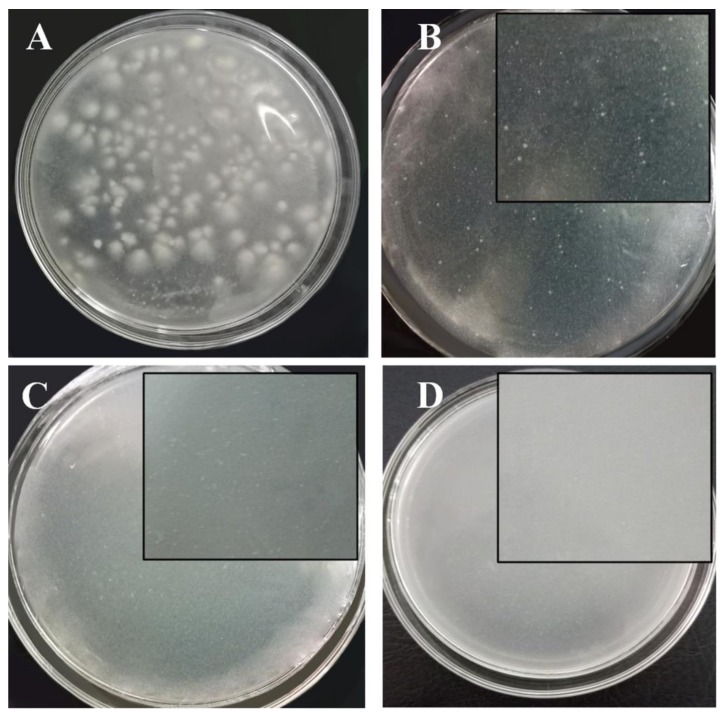
The images of the control agar plate (**A**) and agar plates with OCS2/nSiO_2_ solutions at concentrations of 400 mg L^−1^ (**B**), 600 mg L^−1^ (**C**), and 800 mg L^−1^ (**D**).

To identify the lowest concentration of the hybrid material that can inhibit *P. infestans* growth (MIC), OCS2/nSiO_2_ was chosen for implementation in another bioassay test via the agar dilution method with a concentration range of OCS2/nSiO_2_ from 400 to 1800 mg L^−1^. The results indicate that, in comparison with the growth control plates, which are agar plates without an antifungal agent during the 10-day investigation (Figure 7A), except for the plates with OCS2/nSiO_2_ concentrations of 400 and 600 mg L^−1^ (Figure 7B,C), there was no observed fungal growth in the rest of the plates treated with this antifungal agent at concentrations from 800 to 1800 mg L^−1^; that is, the concentration of 800 mg L^−1^ was the lowest concentration of the OCS2/nSiO_2_ material to completely prevent fungal development (Figure 7D).

Interestingly, although the OCS2/nSiO_2_ concentrations of 400 and 600 mg L^−1^ cannot produce an effective antifungal effect against *P. infestans*, the colonies in the plates with antifungal agent concentrations of 400 and 600 mg L^−1^ are obviously smaller than those in the growth control plates. This result requires further study in future reports.

A study on antifungal activity of penicillic acid isolated from *Aspergillus sclerotiorum* (Filamentous fungi) against *Phytophthora* species was investigated [29]. The results indicated that the minimum inhibitory concentrations (MICs) of this kind of material against *Phytophthora* species were from 5 to 35 µg/disc. Notably, the MIC of penicillic acid inhibiting the growth of *P. infestans* was 5 µg/disc. This pattern was 80 times lower than that of the OCS2/nSiO_2_ material used to inhibit growth of *P. infestans* in this report.

Even though the antifungal activity of the hybrid material in this research was not as effective as those of penicillic acid, it is still considered a potential eco-friendly agrochemical for developing green agriculture field and reducing harmful impacts on the environment and human health caused by toxic fungicides and pesticides. Simultaneously, the novel nontoxic hybrid material can be a new vaccine for stimulating self-defense and growth of plants. Previous reports indicated that the materials from OCS and/or nanosilica created positive impacts on plants physiology and defense response as well as yield increases [11,12]. In contrast, it was found that penicillic acid is cytotoxic and hepatotoxic as well as acutely toxic to animals such as poultry, rats, and rabbits. Furthermore, it is claimed to be carcinogenic and cardiotoxic. It also causes blood vessel dilatability, according to a previous report [30].

**Figure 8:**
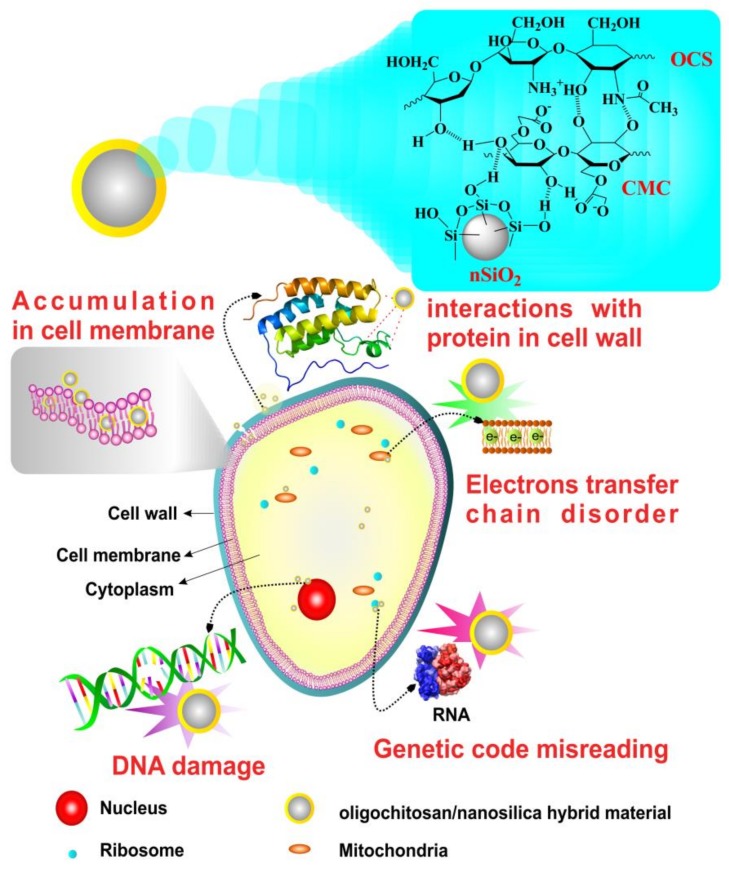
Possible schematic mechanism of the antifungal activity of the OCS-nSiO_2_/CMC hybrid materials.

In this study, the possible antifungal mechanisms of OCS-nSiO_2_/CMC hybrid materials are described (Figure 8). These materials may possess synergistic characteristics of both components, nSiO_2_ and OCS, as mentioned above. nSiO_2_ with tiny size coated by OCS/CMC with active functional groups probably easily deactivate protein molecules in the cell wall and accumulate in the cell membrane, leading to cell lysis. Moreover, the normal physiological functions of a fungal pathogen can be directly disrupted via DNA damage, genetic code misreading, as well as electron transfer chain disorders by the OCS-nSiO_2_ hybrid material, when it penetrates inside the fungal pathogen.

The successful synthesis of new nanostructured hybrid materials with a synergistic effect of the antifungal activities of both nSiO_2_ and OCS is of great importance in developing a new platform for further research with respect to finding environmentally friendly, biodegradable, and natural fungicides. These materials can be considered potential candidates for application in the agriculture field, and can be a substitute for toxic and unhealthy commercial agrochemicals for the sake of a greener world.

## 4. Conclusions

This study shows that novel hybrid materials with a 1:1 (*w*/*w*) ratio of OCS to nSiO_2_ in the presence of 0.3% CMC can be successfully generated. The materials have good solution stabilities of more than one month without aggregation. OCS2/nSiO_2_ (4.21 kDa) was the most stable mixture in comparison with OCS3/nSiO_2_ (3.60 kDa) and OCS1/nSiO_2_ (5.48 kDa). Their particle sizes were 4–8, 2–8, and 3–7 nm, respectively. The possible schematic mechanisms of the antifungal activity of the OCS-nSiO_2_/CMC hybrid materials were also discussed.

All the oligochitosan, silica, and hybrid material samples had good antifungal abilities against *P. infestans*, which causes late blight disease, yet the antifungal abilities of the hybrid materials, due to a synergistic effect, had better antifungal capacities than that of each individual component. Interestingly, the diameters of the inhibition zones of OCS/nSiO_2_ were approximately 4–5 and 7–9 mm larger than those of OCS and nSiO_2_, respectively. Notably, the minimum concentration at which complete inhibition of fungal growth occurred was 800 mg L^−1^, which was a 1.5-fold lower concentration in comparison with that of OCS or nSiO_2_. However, the antifungal ability of OCS2/nSiO_2_ was still very effective. The minimum concentration of OCS2/nSiO_2_ needed for complete *P. infestans* growth inhibition during the 10-day investigation by the agar dilution method was 800 mg L^−1^. We can conclude that these materials have great potential as eco-friendly candidates with excellent fungal resistant activities for use in agriculture to replace current toxic fungicides.

## Figures and Tables

**Table 1:** The average diameter (mm) of the inhibition zones of the antifungal agents at different concentrations against *P. infestans*.

Concentration of Antifungal Agent (mg L^−1^)	The Average Inhibition Zone Diameter (mm)			
	CTS1	nSiO_2_	OCS1	OCS2
600	0.00 ± 0.00	0.00 ± 0.00	0.00 ± 0.00	0.00 ± 0.00
800	0.00 ± 0.00	0.00 ± 0.00	0.00 ± 0.00	0.00 ± 0.00
1000	0.00 ± 0.00	0.00 ± 0.00	0.00 ± 0.00	0.00 ± 0.00
1200	6.83 ± 0.17	7.77 ± 0.15	0.00 ± 0.00	0.00 ± 0.00
1400	7.17 ± 0.17	8.00 ± 0.00	8.33 ± 0.33	9.77 ± 0.39
1600	6.83 ± 0.17	8.83 ± 0.33	10.67 ± 0.93	10.17 ± 0.17
1800	6.67 ± 0.17	9.77 ± 0.15	13.83 ± 0.33	12.00 ± 0.29
**Concentration of** **Antifungal Agent (mg L^−1^)**	**OCS3**	**OCS1/nSiO_2_**	**OCS2/nSiO_2_**	**OCS3/nSiO_2_**
600	0.00 ± 0.00	0.00 ± 0.00	0.00 ± 0.00	0.00 ± 0.00
800	0.00 ± 0.00	10.17 ± 0.17	9.67 ± 0.17	8.00 ± 0.50
1000	0.00 ± 0.00	11.83 ± 0.33	11.50 ± 0.29	10.00 ± 0.76
1200	0.00 ± 0.00	12.67 ± 0.17	12.33 ± 0.33	10.50 ± 0.50
1400	10.17 ± 0.44	13.97 ± 0.30	13.67 ± 0.17	11.67 ± 0.67
1600	13.33 ± 0.88	15.17 ± 0.60	14.83 ± 0.17	13.83 ± 0.44
1800	14.00 ± 1.53	17.17 ± 0.60	16.17 ± 0.44	14.83 ± 0.44
